# Negation detection in Swedish clinical text: An adaption of NegEx to Swedish

**DOI:** 10.1186/2041-1480-2-S3-S3

**Published:** 2011-07-14

**Authors:** Maria Skeppstedt

**Affiliations:** 1 Department of Computer and Systems Sciences (DSV), Stockholm University, Forum 100, SE-164 40 Kista, Sweden

## Abstract

**Background:**

Most methods for negation detection in clinical text have been developed for English text, and there is a need for evaluating the feasibility of adapting these methods to other languages. A Swedish adaption of the English rule-based negation detection system NegEx, which detects negations through the use of trigger phrases, was therefore evaluated.

**Results:**

The Swedish adaption of NegEx showed a precision of 75.2% and a recall of 81.9%, when evaluated on 558 manually classified sentences containing negation triggers, and a negative predictive value of 96.5% when evaluated on 342 sentences not containing negation triggers.

**Conclusions:**

The precision was significantly lower for the Swedish adaptation than published results for the English version, but since many negated propositions were identified through a limited set of trigger phrases, it could nevertheless be concluded that the same trigger phrase approach is possible in a Swedish context, even though it needs to be further developed.

**Availability:**

The triggers used for the evaluation of the Swedish adaption of NegEx are available at http://people.dsv.su.se/~mariask/resources/triggers.txt and can be used together with the original NegEx program for negation detection in Swedish clinical text.

## Background

Medical documentation, such as patient records, is today often stored in a digital, searchable format. This opens the possibility of extracting information, which for example could be used for disease surveillance or to find new, unknown connections between patients’ backgrounds, symptoms and diseases. When extracting information from a text, it is not only the words that occur in the text that are important, but also whether these words are negated or not. This is especially true when it comes to patient records, since when describing the status of a patient, the physician often reasons by excluding various possible diagnoses and symptoms. Many systems, both based on knowledge engineering methods and machine learning methods, have therefore been developed for detecting negated concepts in clinical text.

A basic knowledge engineering system for negation detection in English clinical text, developed by Chapman et al., is the NegEx algorithm. NegEx, which showed a precision of 84.5% and a recall of 82.4% when evaluated on discharge summaries, detects negated findings and diseases through the use of three lists of negation trigger phrases [1]. NegEx was also evaluated on ten other types of clinical texts, which resulted in an average precision of 97% [2], whereas an evaluation on pathology reports, showed a precision that varied from 84% to 19% depending on the section of the report [3].

A system constructed by Elkin et al. [4], was built on the same idea as NegEx, but the method for determining the scope of the negation trigger was extended. The sentences were divided into smaller segments using a list of operators, and a list of words stopping the propagation of the negation trigger was also used. A precision of 91.2% and recall of 97.2% was achieved when the system was used for identifying negated concepts in health records. It was also concluded that the accuracy of the negation detection system varied across the different sections of the health record. Also Mutalik et al. [5] used a set of words that limited the scope of a negation trigger, including personal and relative pronouns as well as conjunctions. In addition to that, the negation triggers were divided into categories, for example based on whether they negate multiple concepts or not. Their system achieved a precision of 91.8% and a recall of 95.7%. Another example of a knowledge engineering approach, but where part-of-speech information was also used, is described by Huang and Lowe [6]. A set of negation triggers and manually constructed grammar rules were used to detect negations in radiology reports, which resulted in a system with a precision of 98.6% and a recall of 92.6%.

NegEx has been extended through another knowledge engineering algorithm called Context, which apart from detecting negations also detects historical and hypothetical clinical conditions, as well as whether a condition is experienced by someone other than the patient. [7]

The negation trigger *not* had a lower precision than the other triggers in the NegEx system. NegEx was therefore also extended through a Naive Bayes classifier and a decision tree, which were used to detect when the trigger *not* indicated a negation. Both these methods, which used features such as surrounding words and their part-of-speech, resulted in an increased precision. [8]

Different machine learning methods for negation detection were also developed and evaluated by Morante and Daelemans [9], as well as by Rokach et al. [10] and Uzuner et al. [11]. For clinical text, Morante and Daelemans achieved a recall of 98.1% for the detection of negation triggers, and a maximum precision of 93.8% when the scope of these triggers was detected through a support vector machine. Rokach et al. used automatically derived regular expressions as attributes in cascaded decision trees, which resulted in a negation detection system with a precision of 94.4% and a recall of 97.4%. Uzuner et al. compared the performance of an extended version of NegEx with a support vector machine system, using features including the four words preceding or following a disease or symptom. The machine learning system achieved higher results, also when trained on one of type of clinical text and tested on another.

Most work on negation detection in medical language has been carried out for English, and there is therefore a need to evaluate the feasibility of adapting those methods to clinical text written in other languages. The objective of this study was therefore to adapt a method for English negation detection to Swedish clinical text, and to evaluate the performance on Swedish compared to English. The hypothesis was that the results for Swedish would be similar to the results for English, since the two languages are grammatically close.

## Methods

Since a basic negation detection method like NegEx shows relatively good results for English, it was considered to be the most suitable system to adapt for this initial study on negation detection in Swedish clinical text. An adaption of NegEx to Swedish and an evaluation of the results compared to the English version of NegEx could give an indication of whether it is necessary to adapt more complex methods for negation detection into Swedish. The results of this study could also be used as a baseline for comparing the results of other methods.

### The NegEx algorithm

NegEx detects *pertinent negatives* in English patient records, that is ”findings and diseases explicitly or implicitly described as absent in a patient”. Given a sentence and a chosen proposition in this sentence, NegEx determines if that proposition is negated or not. An example would be *”Extremities showed no cyanoses.”*, in which the proposition is *cyanoses*. [1]

The NegEx algorithm uses regular expressions and three lists of trigger phrases. The first list, the pre-negation list, consists of trigger phrases which indicate that a proposition that follows them is negated in the sentence, for example *no signs of*. The second list, the post-negation list, consists of trigger phrases that indicate that a proposition preceding them is negated, as the phrase *unlikely*. Finally, the third list consists of pseudo-negation phrases, phrases that are similar to negation triggers, but that do not trigger negation, for example *not certain if*. The algorithm judges the proposition to be negated if it is in the range of one to six words from a post- or pre-negation trigger. [1]

NegEx has later been further developed into NegEx version 2 [12], for example through the addition of more triggers and by limiting the scope of the negation trigger through a list of conjunctions.

In the evaluation of NegEx, the propositions consisted of UMLS phrases that belonged to any of the UMLS categories *finding*, *disease or syndrome *or* mental or behavioral dysfunction* and that could also be found in the describing text of an ICD-10 code. Sentences containing these UMLS phrases were extracted from discharge summaries. Thereafter, 500 of the extracted sentences that contained at least one negation trigger and 500 sentences that did not contain a negation trigger were randomly selected. The sentences were then categorised by physicians into containing an *affirmed proposition*, a *negated proposition* or an *ambiguous proposition*. The inter-rater agreement was almost 100%. For the NegEx evaluation, the categories *affirmed* and *ambiguous* were grouped into the category *not negated*.

As previously mentioned, the results showed a precision of 84.5% and a recall of 82.4% for sentences in the group with negation triggers and a negative predictive value of 97.0% for sentences in the group without triggers. Of the correctly found negations, 82% were triggered by only three negation triggers; *no*, *without* and *no evidence of*. Moreover, only 15 of the 35 negation triggers were found in the test set [1].

### Translation and adaption method

The list of Swedish negation trigger phrases was obtained through a translation of the English negation triggers from *NegEx version 2 *[12]. The translations were made with the help of a web-based English-Swedish dictionary [13] and with the help of Google translate [14]. In the cases where there was a good translation neither in the dictionary nor in the Google translation, the negation was translated by the author of this article. When it was not possible to find a good Swedish translation, the phrase was omitted. A total of 148 phrases were translated. Almost all negation phrases were general English terms. However, in a few cases they consisted of specific medical terms, and in these cases the translation was made by a physician. In many instances, the dictionary offered many translations, and in other cases the same translation was offered for different English phrases. In the cases where several translations were offered, all of them were added to the list of Swedish negations.

English and Swedish are both Germanic languages [15] and they have a similar grammar. Nevertheless, there are some grammatical differences, of which the following had to be taken into account through an expansion of the list of translated trigger phrases.

Swedish has two grammatical genders (common gender and neuter gender), whereas the English language lacks grammatical gender. Adjectives and some quantifiers in Swedish have a gender concord, as well as a number concord [16]. To compensate for this, the English negative quantifier *no* was translated into three different forms of the corresponding Swedish negative quantifier, namely *inga*, *ingen* and *inget*. Inflections of all adjectives were also generated. This was accomplished by invoking the Granska inflector [17,18].

The English combinations of aspect and tense do not always correspond directly to a Swedish verb form [16]. Therefore, a direct translation of the different forms of a verb in the trigger phrase list was not performed. The lemma form of the verb was instead added to the list of negation triggers in Swedish, and from this all inflections of the verb were generated, again using the Granska inflector.

Swedish has a word order inversion in subordinate clauses. The position of the negating adverb is changed, and it is instead positioned immediately before the verb [19]. When stressing the negation, there is also the possibility of using this word order in the main clause [20]. A version with reversed word order was therefore generated for trigger phrases containing some of the most common adverbs. From the translation of the trigger phrase *has not*, a version with the word order *not has* was for example generated.

The difference connected with the do-construction did not need to be taken into account. When negating a non-auxiliary verb in English, the do-construction is used. This type of construction does not exist in Swedish. The phrase *de vet* (*they know*) would for example be negated as *de vet inte* (*they know not*) [21]. However, the NegEx algorithm only checks if the proposition is less than six words to the right of the word *inte* (*not*), and when it is, it will consider the proposition to be negated. The lack of a do-construction should therefore not affect the results.

In order to determine which of the triggers in the expanded trigger list to use for this study, the frequency of each trigger was counted on a text other than the test set, and the most frequent triggers were selected. The number of selected triggers was six more than used in the English NegEx evaluation, to compensate for grammatical differences.

### Evaluation method

Propositions to use for evaluating the performance of the Swedish version of NegEx were taken from the Swedish translation of the ICD-10 codes, the International Classification of Diseases [22]. However, the description in the ICD-10 codes often contains both the name of a symptom or disease and a clarification or specification of it, which has the effect that some of the most common symptoms and diseases would not be found through simple string matching. An automatic pre-processing of the ICD-10 code list was therefore made, where for example text within parenthesis and clarifications such as *not specified* or *other specified forms* were removed. Additional lists were also added to the proposition list, including the KSH97-P [23], an adaption of the ICD-10 codes for primary care, and the MeSH terms under the sections *diseases* and *mental disorders*.

The test data was extracted from a set of sentences randomly chosen from the assessment field from different parts of Swedish health records in the Stockholm EPR Corpus [24]. From this set, sentences that contained any of the diseases or symptoms in the proposition list were extracted, even if the proposition was part of a compound word. The chosen sentences were ordered in a list of pairs, consisting of the sentence and the proposition. If a sentence contained more than one proposition, the sentence was added to the list once for each proposition.

In order to be able to compare the English and Swedish versions of NegEx, the same evaluation method was used, and two groups of test sentences were constructed. The first group contained 558 sentences with at least one of the trigger phrases, and the second group contained 342 sentences without any of the trigger phrases.

The propositions were manually classified into the categories *affirmed*, *negated* and *ambiguous*. The class *ambiguous* was defined as either one of the following: The author was uncertain whether the patient had the symptom or disease, it could not be determined from the sentence whether the patient had the symptom or disease, the mentioned symptom or disease did not refer to the patient, or the statement did not refer to a present condition.

The group of sentences containing negation triggers was classified by two physicians, one of them classifying 70 sentences, and the other classifying the remaining 488 sentences. As a reference, all sentences were also classified by a rater without medical education, who henceforth will be called the *non-medical expert*, and the inter-rater agreement between this classifier and the physicians was measured. The classifications made by the physicians were used for the gold standard.

In the group of sentences without negation triggers, the non-medical expert classified all sentences, and one of the physicians classified 95 sentences. Also 35 sentences that were subjectively judged by the non-medical expert as not possible to be rated without deep medical knowledge, were rated by the physician. When there existed a classification made by the physician, that classification was used for the evaluation, and in the other cases the classification made by the non-medical expert was used. For the 95 sentences that were classified by two raters, the inter-rater agreement was measured. The inter-rater agreement for both groups of sentences is presented in the results section.

As in the evaluation of the English NegEx, the categories *affirmed* and *ambiguous* were collapsed into the category *not negated*. The results of the classification are presented in Table 1.

**Table 1:** Results of the classification

	Negated	Not negated	Total
Sentences with negation triggers	269	289	558
Sentences without negation triggers	12	330	342

Number of sentences manually classified as *negated* and *not negated*.

The Swedish version of NegEx was then executed separately with the sentences containing negation triggers, and the sentences not containing negation triggers, as input. The NegEx system [25] could be used in a Swedish context, together with the constructed trigger list [26], without any major modifications. Precision, recall, specificity and negative predictive value were measured using the manually classified sentences as a gold standard. Precision and frequency of each negation trigger were also measured.

In order to evaluate whether there were any common Swedish trigger phrases for negation that were not obtained through the translation of the English triggers, the translated list was compared to a list of manually annotated negation triggers. This list was derived from a Swedish clinical text in which triggers for uncertainty and negation had been manually annotated in a study carried out by Dalianis and Velupillai [27].

The research was carried out after approval from the Regional Ethical Review Board in Stockholm (Etikprövningsnämnden i Stockholm), permission number 2009/1742-31/5.

## Results

For the group of sentences that contained negations triggers, the precision was 75.2% and the recall was 81.9%, as shown in Table 2. The group containing sentences without trigger phrases showed a negative predictive value of 96.5%. The recall and negative predictive value were thus almost identical for English and Swedish, whereas the precision was lower for the Swedish version. The significance of the difference in precision between Swedish and English was measured using the *χ*^2^-test, which showed that it was significantly lower for Swedish (p < 0.01).

**Table 2:** Results for the Swedish adaption of NegEx

Sentences with negation trigger phrases	English (%)	Swedish (%) (95% CI)
Recall (sensitivity)	82.4	81.9 (77.3 - 86.4)
Specificity	82.5	74.7 (69.6 - 79.7)
Precision (positive predictive value)	84.5	75.2 (70.2 - 80.1)
Negative predictive value	80.2	81.4 (76.7 - 86.1)
		
**Sentences without negation trigger phrases**	**English (%)**	**Swedish (%) (95% CI)**

Negative predictive value	97.0	96.5 (94.5 - 98.6)

*Recall*: No. of correctly detected negated propositions divided by no. of manually rated negated propositions. *Specificity*: No. of propositions correctly detected as not negated divided by no. propositions that were manually rated as not negated. *Precision*: No. of correctly detected negated propositions divided by total no. of propositions that NegEx classified as negated. *Negative predictive value*: No. of propositions that NegEx correctly did not classify as negated divided by total no. of propositions that NegEx did not classify as negated. (Figures for English from Chapman et al. [1].)

The precision and frequency of each trigger are shown in Table 3. The three most common triggers were the common gender form and the plural form of no (*ingen*, *inga*), and the trigger *not* (*inte*). Together, they are the trigger phrase in 64.9% of the correctly identified negations. Including the phrase in fourth place, no signs of, they trigger 75.7% of the negated propositions that were correctly identified.

**Table 3:** The most frequent triggers

Phrase		Number of occurrences	Precision
förnekar	(denies)	3	100,0%
aldrig	(never)	3	100,0%
avsaknad av	(absence of)	2	100,0%
inga tecken	(no signs of)	25	96,0%
ingen	(no, common gender)	84	90,5%
inga	(no, plural)	48	87,5%
inget	(no, neuter gender)	6	83,3%
inte har	(not have)	6	66,7%
utan tecken	(without signs of)	3	66,7%
utan	(without)	20	65,0%
inte	(not)	45	57,8%
ej	(not)	31	54,8%
inte visar	(does not show)	6	50,0%
har inte	(have not)	4	50,0%
icke	(non-, not)	7	0,0%

All triggers that occur more than once, their precision and the number of times they occur in the sentences.

In the test set, only 18 of the 41 trigger phrases in the trigger list were found. None of the post-negation triggers and two pseudo-negations were found in the Swedish test data. Of the negation triggers, 14 correctly negated a proposition.

### Inter-rater agreement

In the group of sentences without negation triggers, there was 100% agreement for the 95 sentences that were classified by both the physician and the non-medical expert. None of these sentences were classified as negated.

In the group of sentences with a negation trigger, on the other hand, the inter-rater agreement between the physicians and the non-medical expert with respect to the two groups *negated* and *not negated* was 87.4%. Cohen’s Kappa with respect to these two classes was 0.745. Of the sentences where there was a disagreement, 72% had received the classification *ambiguous* by one of the raters before the groups *affirmed* and *ambiguous* were collapsed into one group. The non-medical expert classified more sentences as *ambiguous* than the two physicians, 99 sentences compared to 71.

When instead using the classification made by the non-medical expert for the evaluation of the sentences containing negation triggers, the precision was 69.0% and the recall was 89.9%.

## Discussion

The lower precision for the Swedish adaption could perhaps partly be explained by the different types of clinical text. The English version was evaluated on discharge summaries, whereas the Swedish version was evaluated on the assessment field, which possibly contains more reasoning and therefore perhaps more uncertain expressions. This is supported by the fact that 20% of the sentences that were incorrectly classified by NegEx as negated were rated as ambiguous by the annotator. These sentences all contained phrases expressing uncertainty, such as *no evident*, *not certain* or *no real noticeable*. That the performance of an algorithm such as NegEx is affected by the type of clinical text is also supported by many of the previously mentioned studies.

Another source of error was the trigger *icke* (*non-*, *not*), since it is a common construction for a name of a disease, or a version of a disease, to have a name that starts with this word, for example *icke allergisk astma* (*non-atopic asthma*). The disease is thus present in the patient, even though the word *icke* is interpreted as a negation trigger by NegEx. In the test data, all occurrences of the word *icke* were constructions like this, constituting 10% of the incorrectly negated sentences. Also the Swedish word for *without* (*utan*) was a problematic trigger phrase, since *utan* is also a conjunction meaning *but*. This trigger gave rise to 5% of the instances where a proposition was incorrectly classified as negated. Removing the trigger *icke* and implementing a regular expression disambiguation rule, based on the observation that the Swedish conjunction *utan* very often is preceded by the word *not *(*inte*) earlier in the sentence, resulted in a precision of 77.9%.

Other error types were also identified. These were, however, not specific to Swedish or the type of test data, and could therefore not account for the difference in precision between the English and Swedish versions of NegEx. Examples are when the negation of the proposition occurred in a conditional clause, which was the case for 10% of the incorrectly classified sentences, or when the scope of the trigger should be less than the NegEx scope of six words, which was the case for 5% of the incorrectly classified sentences.

Of the incorrectly affirmed sentences, 27% said that the patient used to have a disease or symptom and 12% were hypothetical or conditional sentences. In 16% of the incorrectly affirmed sentences, the negation trigger was more than six words from the proposition and in 14% of the sentences, a pre-negation trigger was after the proposition.

### Completeness of the used trigger list

No common negation triggers that were not in the trigger phrase list were found in the test data. The only re-occurring trigger that was not included in any of the three lists were two other forms of the phrase *rule out* than what was included in the trigger list.

Nor did the derived list of manually annotated triggers, which is shown in Table 4, contain any frequent negation triggers that were not obtained through the translation of English triggers. However, two less common re-occurring manually annotated triggers, both meaning *nothing*, were not included. The method of translating English triggers was thus not sufficient for finding some of the more unusual Swedish phrases.

**Table 4:** Manually annotated negation triggers

Phrase		Annot. 1	Annot. 2	Annot. 3
inte	(not)	307	368	277
ingen	(no, common gender)	227	255	218
ej	(not)	176	186	177
inga	(no, plural)	164	177	159
inget	(no, neuter gender)	51	55	47
utan	(without)	34	63	39
icke	(non-, not)	8	4	6
ingenting	(nothing)	4	3	3
intet	(nothing)	2	1	1
aldrig	(never)	0	0	2

All negation triggers that occurred more than once in the manually annotated clinical text described by Dalianis and Velupillai. In their study, 6 740 sentences were manually annotated for negation and uncertainty by three different annotators. [27]

The two negation triggers *förnekar* (*denies*) and *avsaknad av* (*absence of*) were obtained through translation of English triggers, but not through the manual annotation of negation triggers. This indicates that to find less common negation triggers, it is not always enough to scan through a large number of sentences, but that other methods for finding triggers might also be needed.

### Comparison between the English and Swedish trigger phrases

There are many similarities between the results for the Swedish and the English evaluations. In both languages, there are a small number of negation triggers that are very common in the evaluation sentences, and the rest of them only occur a few times or not at all. It can also be noted that both in Swedish and English, the precision of the trigger *not* (*inte*) is low.

The similarities, and the fact that almost all common triggers that were manually annotated, were obtained through a translation of English triggers, indicate that negations are constructed in a similar way in English and Swedish health records, and that the approach with a limited set of trigger phrases is also possible to use on Swedish clinical text.

### Limitations

The most important limitation of this study was the relatively low inter-rater agreement with respect to sentences that could be classified as either negated or ambiguous. This resulted in a lower precision when the adapted NegEx instead was evaluated against the classifications made by the non-medical expert. It is not unlikely that the non-medical expert classified more sentences as ambiguous because of less familiarity with the style of writing in clinical text. However, since no inter-rater agreement study between the two physicians was made, it could also be the case that the instructions for classifying were interpreted differently by the two groups of raters, independently of their difference in medical background. Therefore, the relatively low inter-rater agreement adds uncertainty to the results.

In the original NegEx study, a few sentences that contained phrases that were suspected to sometimes indicate a negation, but that were not in the three trigger lists, were also included in the group of sentences with negation triggers. No such sentences were included for the evaluation of the Swedish version, and this might have resulted in a slightly higher recall for Swedish. Also the fact that a wider range of propositions were used for the Swedish evaluation, could be a relevant limitation of the comparison, as well as the previously mentioned differing types of clinical texts.

Another limitation is that neither the pre-processing of the ICD-10 code list, nor the detection of a proposition in a compound word was perfect, which had the effect that some test sentences did not contain a symptom, disease or finding. These sentences were therefore manually removed from the test data by the raters. In 28 cases, there was a disagreement between the two raters whether a sentence should be included or not, and these sentences were not included in the inter-rater agreement calculations. Also, as in the study by Chapman et al. [2], no analysis was made of the occurrences of negations that stretch over sentence boundaries.

Negation was defined to also include temporality, that is medical conditions that the patient only experienced in the past were defined as negated, and conditions that were negated in the past were defined as ambiguous. Also, negations in conditional phrases and negations that were expressed with uncertainty were classified as ambiguous, and thereby as *not negated* in the gold standard. An evaluation of negation in isolation would therefore also have been relevant, where anything that was negated, whether it was historical, uncertain or hypothetical, was classified as negated in the gold standard. This definition would give higher precision and recall, and also facilitate the comparison with other negation detection studies.

### Future work

The Swedish negation detection could probably be improved through the use of more advanced natural language processing methods, for example through using the output of a syntactic parser to determine the scope of a negation trigger.

The two forms of *rule out* that triggered negation in the test data, but that were not among the 41 Swedish negation triggers used for this study, were included in the complete list of translated trigger phrases. To use the complete list of negation triggers would thus slightly increase in the number of negated concepts that were detected. It would therefore also be interesting to evaluate on a larger test set how a use of the complete trigger list would affect precision.

The results indicate that to automatically distinguish an ambiguity from a negation is the most difficult part of negation detection. Therefore, to improve the negation detection, it probably needs to be combined with detection of uncertainty. Also, the aspects investigated in the Context study [7], such as detection of the experiencer of a medical problem and the temporality of the problem, need to be addressed.

The relatively low inter-rater agreement with respect to concepts that might be classified as either ambiguous or negated, indicates that it is a difficult task also for a human rater to determine whether a statement is an ambiguity expressed as a negation or an actual negation. This needs to be further studied, for example through the development of a richer set of classes and more detailed guidelines for the classification. As mentioned above, in order to facilitate a comparison with other negation detection studies, an evaluation is also needed of the performance of the Swedish NegEx when all negated sentences are included in the negated class, regardless if they are historical, uncertain or in a conditional phrase.

## Conclusions

The Swedish version of the NegEx algorithm had a significantly lower precision than the English version, and the recall was almost identical for the English and Swedish versions. Not taking the uncertainty of the low inter-rater agreement into account, the Swedish version has a precision of 75.2% and a recall of 81.9% for sentences containing the trigger phrases and a negative predictive value of 96.5% for sentences not containing any trigger phrases. As with the English version, a small number of trigger phrases accounted for the majority of the detected negations.

Since a limited set of triggers can be used to identify many negations also in Swedish, the simple approach of the NegEx algorithm can be used as a base method for identifying negations in Swedish. However, even for use in a system without high demands on robustness, the method needs to be further developed.

## Competing interests

The author declares that she has no competing interests.
